# Pheomelanin Effect on UVB Radiation-Induced Oxidation/Nitration of l-Tyrosine

**DOI:** 10.3390/ijms23010267

**Published:** 2021-12-27

**Authors:** Alessia Mariano, Irene Bigioni, Anna Scotto d’Abusco, Alessia Baseggio Conrado, Simonetta Maina, Antonio Francioso, Luciana Mosca, Mario Fontana

**Affiliations:** 1 Department of Biochemical Sciences, Sapienza University of Rome, Piazzale Aldo Moro 5, 00185 Rome, Italy; alessia.mariano@uniroma1.it (A.M.); bigioni.1639690@studenti.uniroma1.it (I.B.); anna.scottodabusco@uniroma1.it (A.S.d.); simonetta.maina@yahoo.it (S.M.); antonio.francioso@uniroma1.it (A.F.); luciana.mosca@uniroma1.it (L.M.); 2 National Heart & Lung Institute, Imperial College London, Norfolk Place, London W2 1PG, UK; a.baseggio-conrado@imperial.ac.uk

**Keywords:** pheomelanin, nitrotyrosine, dityrosine, photooxidation, photosensitizer

## Abstract

Pheomelanin is a natural yellow-reddish sulfur-containing pigment derived from tyrosinase-catalyzed oxidation of tyrosine in presence of cysteine. Generally, the formation of melanin pigments is a protective response against the damaging effects of UV radiation in skin. However, pheomelanin, like other photosensitizing substances, can trigger, following exposure to UV radiation, photochemical reactions capable of modifying and damaging cellular components. The photoproperties of this natural pigment have been studied by analyzing pheomelanin effect on oxidation/nitration of tyrosine induced by UVB radiation at different pH values and in presence of iron ions. Photoproperties of pheomelanin can be modulated by various experimental conditions, ranging from the photoprotection to the triggering of potentially damaging photochemical reactions. The study of the photomodification of l-Tyrosine in the presence of the natural pigment pheomelanin has a special relevance, since this tyrosine oxidation/nitration pathway can potentially occur in vivo in tissues exposed to sunlight and play a role in the mechanisms of tissue damage induced by UV radiation.

## 1. Introduction

**Figure 1:**
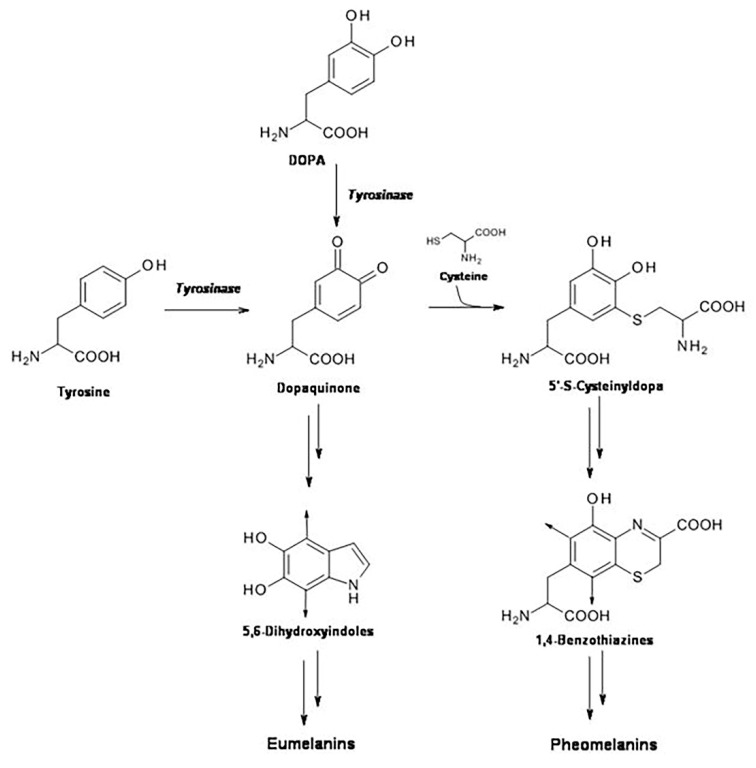
Schematic pathway of eumelanin and pheomelanin biosynthesis.

Pheomelanin is one of the existing forms of the natural pigment melanin. Melanin is present in the skin in two forms: eumelanin and pheomelanin. Eumelanin is a heterogeneous polymer composed mainly of dihydroxyindole units derived from tyrosinase-catalyzed oxidation of tyrosine or 3,4-dihydroxyphenylalanine (DOPA) to dopaquinone. Compared to eumelanin, pheomelanin structure differs due to non-enzymatic addition of cysteine to dopaquinone during the pathway of pigment biosynthesis. DOPA-derivatives with cysteine, such as 5′-S-cysteinyldopa and in minor amount 2′-S-cystenyldopa, are incorporated into the pigment in the form of 1,4-benzothiazine units (Figure 1) [1]. Before being incorporated into pheomelanin, a minor part of 1,4-benzothiazine units may undergo further structural modifications with formation of benzothiazole moiety which copolymerizes with benzothiazine units [2,3,4]. Interestingly, slight variations in the monomer composition of pigment polymer skeleton have been shown to determine significant differences in light absorption, antioxidant activity, redox behavior, and metal chelation [5].

It is commonly believed that melanin plays an important role in the modulation of the photochemical reactions that occur in the skin. Numerous experimental and clinical evidences have shown a protective role of eumelanin on the damage triggered by UV irradiation on the skin [6]. A lower incidence of UV-induced skin diseases is observed in individuals with darker skin pigmentation, where eumelanin is present. Conversely, a higher incidence of UV-induced skin diseases was found in red-haired individuals with pale skin and freckles. Traditionally, this UV susceptibility trait has been associated with a high tendency to sunburn and an increased risk of skin tumors and melanoma [7,8,9]. The damage caused by UV rays would be determined either by the absence of pigmentation or by the photosensitizing properties of the pheomelanin present in the skin of these individuals. Notably, pheomelanin has the capacity to act as photosensitizer by inducing the generation of reactive oxygen species (ROS) upon irradiation with UV light [8,10,11,12,13,14]. Pheomelanin has been observed to increase lipid peroxidation following exposure of liposomes to UV irradiation, suggesting that pheomelanin may act as a pro-oxidant [15]. Analogously to other photosensitizing substances, pheomelanin is able to trigger, following exposure to UV radiation, photochemical reactions capable of modifying and damaging cellular components [16,17]. Furthermore, pheomelanin itself undergoes a photolytic process mediated by the same reactive intermediates [18].

In particular, by exposure to UV radiation, aromatic rings present in the pheomelanin (Pheo) are excited to the singlet state (^1^Pheo*) and rapidly converted to the excited triplet state (^3^Pheo*) [19,20].

The triplet state of pheomelanin can act as photosensitizer triggering photooxidative events by radical-mediated (type I) and singlet oxygen-mediated (type II) mechanisms. The type I mechanism involves free radical formation through the hydrogen atom or electron ransfer by interaction of the triplet excited state of the sensitizer with target molecules (S) or molecular oxygen.

^3^Pheo* + S → Pheo^•−^ + S^•+^

Superoxide anion (O_2_^•−^) is generated when the pigment in the excited triplet state transfers electrons to molecular oxygen by type I mechanism [10,21].

^3^Pheo* + O_2_ → Pheo^•+^ + O_2_^•−^

The type II process involves the generation of singlet oxygen (^1^O_2_) by energy transfer from the excited triplet sensitizer to a ground state oxygen molecule [10,12].

^3^Pheo* + O_2_ → Pheo + ^1^O_2_

Recently, pheomelanin has also been implicated in UV-independent pathways of oxidative stress [22].

In this study, the photoproperties of this natural pigment were studied by analyzing the effect of pheomelanin on the oxidation/nitration of tyrosine induced by UVB radiation under different pH values and in presence of iron ions. In particular, pheomelanin effect on UVB-induced oxidation/nitration of tyrosine has been studied at physiological pH and at a weakly acid pH. Under pathophysiological situations, such as inflammation, tissue pH close to 5.5–6 can be found. Moreover, recent studies have shown that acid melanosomal pH suppress melanogenesis, especially eumelanin formation, in melanocytes [23]. Notably, it has been observed that, at pH 5.8, eumelanin biosynthesis is suppressed, while pheomelanin production is enhanced [24].

**Figure 2:**
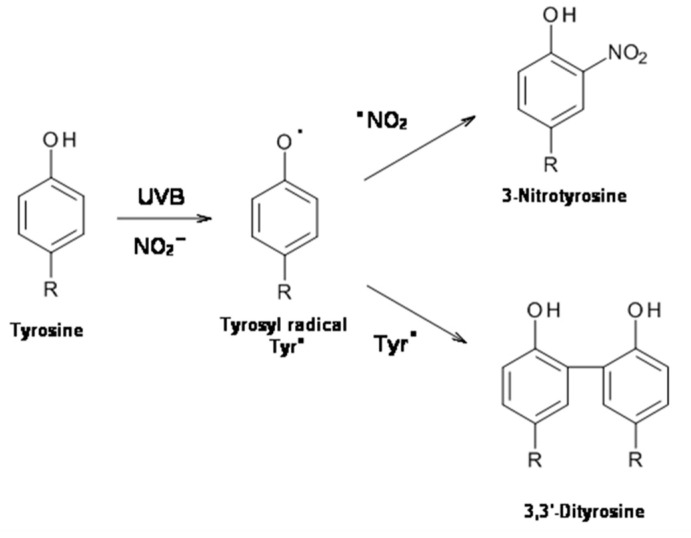
UVB-induced 3-nitrotyrosine and 3,3′-dityrosine formation.

Following UVB radiation of l-Tyrosine, tyrosyl radical that is generated dimerizes with the formation of 3,3′-dityrosine; in presence of nitrite the photochemical reaction produces tyrosyl radical and reactive nitrogen species which combine to form 3-nitrotyrosine as a further product [25,26,27] (Figure 2).

Both 3,3′-dityrosine and 3-nitrotyrosine are considered diagnostic markers of the in vivo production of reactive oxygen and nitrogen species [27,28,29]. Although reactive nitrogen species, such as peroxynitrite, has been the most widely studied nitrating species, tyrosine nitration occurs also through several alternative routes. At this regard, free tyrosine and tyrosine protein residue nitration can be achieved through mechanisms involving peroxidase/H_2_O_2_-dependent oxidation of nitrite to nitrogen dioxide radical (^•^NO_2_) [30,31,32]. In inflammation, myeloperoxidase from activated leukocytes catalyzes tyrosine nitration at high levels [33,34,35,36]. Photonitration of tyrosine to 3-nitrotyorosine has been already shown by methylene blue dye and riboflavin as sensitizers [37,38]. Methylene blue-sensitized photomodification of tyrosine in the presence of nitrite occurs mainly through a process which involves singlet oxygen (type II mechanism). Conversely, singlet oxygen plays a minor role in the tyrosine photooxidation/photonitration mediated by riboflavin as sensitizer [38,39,40]. Interestingly, the oxidation and nitration of tyrosine residues in proteins are considered important post-translational modifications with consequences on the function of proteins and therefore on cellular homeostasis [41,42,43,44].

The study of the photomodification of tyrosine in presence of the natural pigment pheomelanin has a special relevance, since this tyrosine oxidation/nitration pathway can potentially occur in vivo in tissues exposed to sunlight and play a role in the mechanisms of tissue damage induced by UV radiation.

## 2. Results

### 2.1. UVB Radiation-Induced Photooxidation/Photonitration of l-Tyrosine

**Figure 3:**
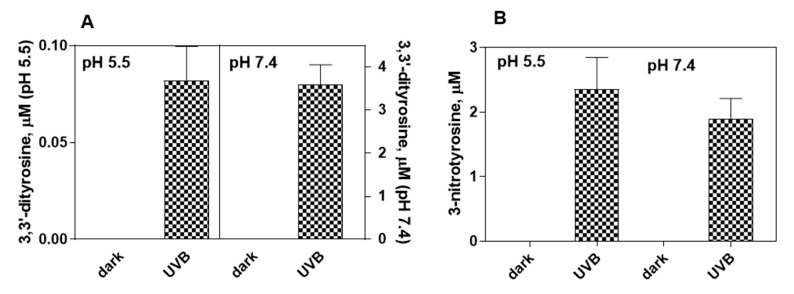
UVB-induced photooxidation/photonitration of tyrosine. A reaction mix containing 1 mM tyrosine in 0.2 M K-phosphate buffer at pH 5.5 or pH 7.4, 0.1 mM DTPA, 10 mM K-nitrite is exposed to UVB radiation. After 30 min of exposure, the reaction is stopped by placing the mixture in the dark and the solution is analyzed by HPLC, to determine the formation of 3,3’-dityrosine (**A**) and 3-nitrotyrosine (**B**), as reported in Materials and Methods. Controls in the dark correspond to the unexposed solution.

The exposure to ultraviolet light (UVB), at room temperature, of a solution containing 1 mM tyrosine leads to the formation of 0.25 ± 0.07 μM at pH 5.5 and 0.13 ± 0.02 μM at pH 7.4 of 3.3’-dityrosine after 30 min of exposure. Tyrosine dimerization was not observed in controls kept in the dark. The exposure of 1 mM tyrosine solution to UVB radiation in the presence of 10 mM nitrite, under the same experimental conditions reported above, leads to 3-nitrotyrosine as a further product in addition to 3,3’-dityrosine (Figure 3). When nitrite is present, 3,3′-dityrosine is 0.08 ± 0.02 μM and 3.60 ± 0.46 μM, at pH 5.5 and pH 7.4, respectively. The amount of 3-nitrotyrosine formed is 2.37 ± 0.4 μM and 1.89 ± 0.15 μM, at pH 5.5 and pH 7.4 respectively, after 30 min of exposure. At low pH values nitrite generates nitrating species which, in the presence of tyrosine, lead to the formation of 3-nitrotyrosine [45]. Control experiments, in which tyrosine and nitrite are incubated in the dark, indicate that, under our experimental conditions, this reaction pathway can contribute minimally to the production of 3-nitrotyrosine only at pH below 3.3.

### 2.2. Effect of Pheomelanin on UVB Radiation-Induced Photooxidation/Photonitration of l-Tyrosine

**Figure 4:**
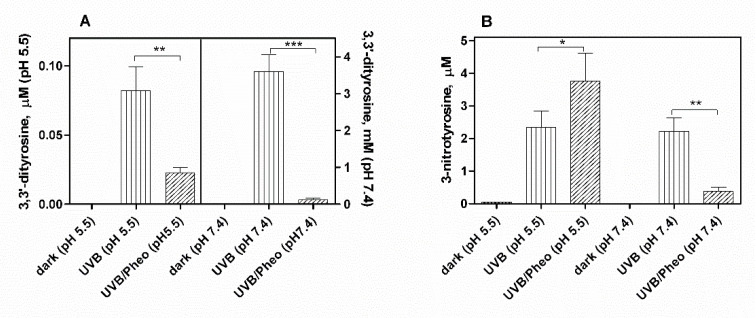
Photooxidation/photonitration of tyrosine by the nitrite/pheomelanin/UVB system. Pheomelanin 4.2 μg/mL is added to reaction mixture containing 1 mM tyrosine, 10 mM K-nitrite, 0.1 mM DTPA in 0.2 M K-phosphate buffer at pH 5.5 or pH 7.4. The solution is exposed to UVB rays for 30 min. The reaction is stopped by placing the mixture in the dark and the supernatant, obtained after centrifugation, is analyzed by HPLC to measure 3,3’-dityrosine (**A**) and 3-nitrotyrosine (**B**), as reported in Materials and Methods. Controls in the dark correspond to unexposed reaction mixtures (pheomelanin/nitrite/tyrosine system).*** *p* < 0.001, ** *p* < 0.01, * *p* < 0.05.

**Figure 5:**
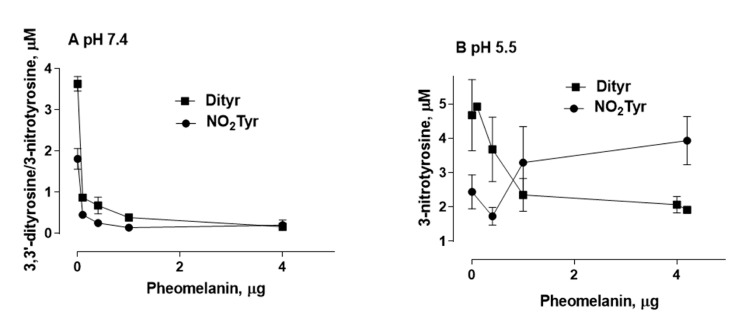
Photooxidation of tyrosine by the nitrite/pheomelanin/UVB system at various synthetic pheomelanin concentrations. Pheomelanin (0.1–4 μg/mL) is added to a reaction mixture containing 1 mM tyrosine, 10 mM K-nitrite, 0.1 mM DTPA in 0.2 M K-phosphate buffer at pH 7.4 (**A**) or pH 5.5 (**B**). The solution is exposed to UVB rays for 30 min. The reaction is stopped by placing the mixture in the dark and the supernatant, obtained after centrifugation, is analyzed by HPLC to determine the formation of 3-nitrotyrosine (●) and 3,3′-dityrosine (■) as reported in Materials and Methods.

In order to evaluate the photoproperties of pheomelanin on the oxidative/nitrative modifications of tyrosine induced by UVB rays, 1 mM tyrosine and 10 mM nitrite were exposed to UVB radiation in the presence of 4.2 μg/mL synthetic pheomelanin at physiological pH 7.4 and pH 5.5. Pheomelanin was enzymatically prepared from l-Dopa and cysteine as reported in the experimental section. After an exposure of 30 min both the formation of 3,3’-dityrosine and the conversion of tyrosine to 3-nitrotyrosine was assayed. Overall, pheomelanin exerts a photoprotective effect (antioxidant) on the oxidation/nitration of tyrosine induced by UVB radiation (Figure 4). However, at pH 5.5 pheomelanin acts as photosensitizer (prooxidant) in the nitrative modification of tyrosine. As shown in the Figure 5B, pheomelanin does not inhibit the nitration of tyrosine but there is a 60% increase in the formation of 3-nitrotyrosine compared to the control exposed to UVB radiation in the absence of pheomelanin. In control experiments in which pheomelanin alone and nitrite were exposed to UVB radiation, neither nitrotyrosine nor dityrosine were detectable.

Figure 5 shows the formation of 3,3’-dityrosine and 3-nitrotyrosine at various concentrations of pheomelanin (0.1–4 μg/mL) at pH 7.4 and pH 5.5. At all concentrations used, pheomelanin has a dose-dependent photoprotective action on the formation of 3,3′-dityrosine at both pH 5.5 and pH 7.4. The photosensitizing action on the formation of 3-nitrotyrosine at pH 5.5 is observed in the range 0.4–4 μg/mL.

### 2.3. Photoproperties of Pheomelanin on UVB-Induced Oxidative/Nitrative Modifications of l-Tyrosine: Effect of Fe(III)

**Figure 6:**
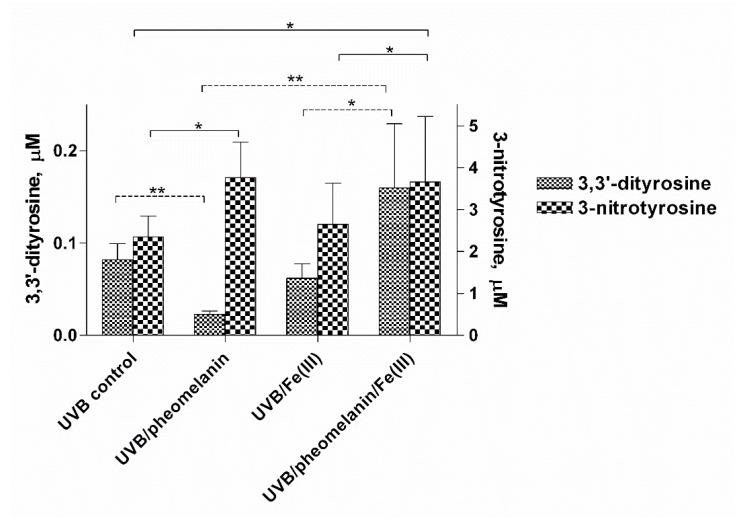
Photooxidation of tyrosine by the nitrite/pheomelanin/UVB system: effect of Fe(III). Pheomelanin 4.2 μg/mL is added to the reaction mixture containing 1 mM tyrosine, 10 mM K-nitrite, 0.1 mM DTPA in 0.2 M K-phosphate buffer at pH 5.5. The UVB control is pheomelanin free. Fe (III) is added as FeCl_3_ at a concentration of 0.1 mM. The solution is exposed to UVB rays for 30 min. The reaction is stopped by placing the mixture in the dark and the supernatant, obtained after centrifugation, is analyzed by HPLC to determine the formation of 3-nitrotyrosine and 3,3′-dityrosine, as reported in Materials and Methods. ** *p* < 0.01, * *p* < 0.05.

It is known that melanins have the ability to bind various metals with the result of modifying their photoproperties [46,47,48,49]. In order to evaluate how the presence of metals can influence the oxidative/nitrative modifications of tyrosine, exposure to UVB rays was performed with the addition of Fe(III) to the reaction mixture. Experiments performed in the absence of metal chelator DTPA showed analogous results (data not shown). At pH 5.5, it is observed that the presence of metals influences the photoproperties of pheomelanin by reducing its antioxidant activity against dityrosine formation (Figure 6). Regarding the formation of 3-nitrotyrosine, the photosensitizer effect of pheomelanin is not affected either by the absence of the chelator or by the addition of Fe(III).

### 2.4. Pheomelanin Effect on Oxidative/Nitrative Modifications of l-Tyrosine Induced by UVB Radiation: Role of Singlet Oxygen

**Figure 7:**
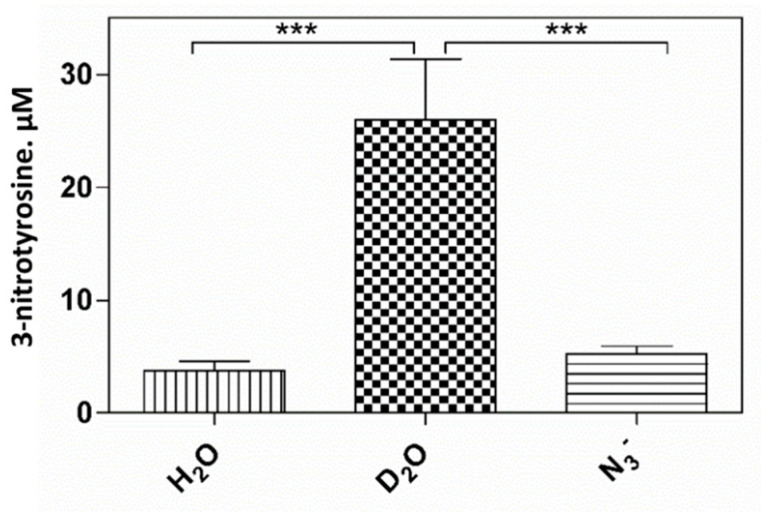
Photooxidation of tyrosine by the nitrite/pheomelanin/UVB system: effect of D_2_O and NaN_3_. Pheomelanin 4.2 μg/mL is added to the solution, containing 1 mM tyrosine, 10 mM K-nitrite in 0.2 M K-phosphate buffer at pH 5.5 and 0.1 mM DTPA. The solution is exposed to UVB rays for 30 min. The reaction is stopped by placing the mixture in the dark and the supernatant, obtained after centrifugation, is analyzed by HPLC to determine the formation of 3-nitrotyrosine, as reported in Materials and Methods. In D_2_O, the pD (5.5) was taken as the measured pH + 0.4. NaN_3_ is added to a final concentration of 1 mM. *** *p* < 0.001.

The photooxidative reactions can be the result of radical type processes (type I) or of processes mediated by singlet oxygen (type II). Both mechanisms can contribute to the photooxidative reactions at the same time. In order to evaluate the role of singlet oxygen (^1^O_2_) in pheomelanin-sensitized nitration reaction of tyrosine at pH 5.5, the yields of 3-nitrotyrosine in H_2_O and D_2_O as solvent were compared. Replacement of H_2_O by D_2_O increases the lifetime of singlet oxygen by about 15 times [50] and, consequently, stimulates ^1^O_2_-dependent reactions. As shown in Figure 7, the production of 3-nitrotyrosine is approximately 8.4 times greater in D_2_O than in H_2_O. This effect is indicative of the participation of singlet oxygen in the reaction. The formation of 3,3’-dityrosine is not affected by D_2_O (Figures S1 and S2).

**Figure 8:**
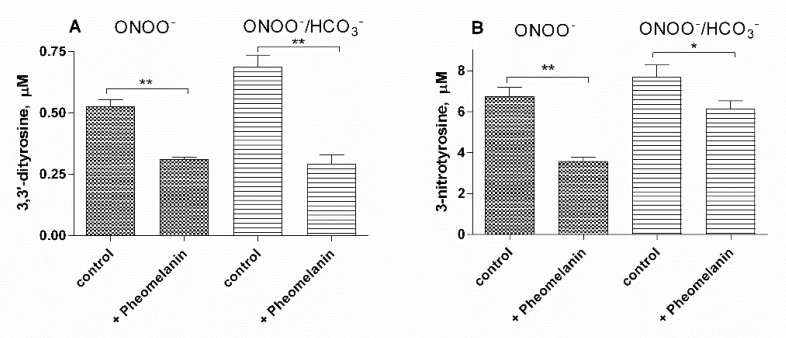
Pheomelanin effect on peroxynitrite-induced oxidation/nitration of tyrosine. To the reaction mix containing 100 µM tyrosine, 4.2 μg/mL pheomelanin, 0.1 mM DTPA in 0.2 M K-phosphate buffer, 100 µM peroxynitrite is added, Na-bicarbonate when present is at a concentration of 25 mM. After 5 min at room temperature, the reaction mixture is analyzed by HPLC to measure 3,3’-dityrosine (**A**) and 3-nitrotyrosine (**B**), as reported in Materials and Methods. ** *p* < 0.01, * *p* < 0.05.

It has been also observed that the formation of 3-nitrotyrosine is significantly reduced in the presence of sodium azide (NaN_3_), a known quencer of singlet oxygen (Figure 8). The inhibitory effect of azide confirms intermediacy of type II mechanism in the pheomelanin-sensitized formation of 3-nitrotyrosine.

### 2.5. Pheomelanin Effect on Oxidative/Nitrative Modifications of l-Tyrosine Induced by Peroxyitrite

Peroxynitrite induces both tyrosine oxidation to 3,3’-dityrosine and tyrosine nitration to 3-nitrotyrosine. Under our experimental conditions, peroxynitrite (100 μM, final concentration) added to a solution containing 100 μM of tyrosine generates 0.53 ± 0.02 μM of 3,3’-dityrosine and 6.75 ± 0.27 μM of 3-nitrotyrosine, respectively. As shown in Figure 8, pheomelanin, at a concentration of 4.2 μg/mL, is able to inhibit both the formation of 3,3’-dityrosine (~42%) and that of 3-nitrotyrosine (~47%). As reported [51,52], peroxynitrite reacts, in vivo, mainly with carbon dioxide, forming a peroxynitrite-CO_2_ adduct which decomposes generating the nitrogen dioxide radicals (^•^NO_2_) and carbonate radical anion (CO_3_^•−^). In the presence of bicarbonate, tyrosine nitration mediated by peroxynitrite is generally increased due to high oxidative/nitrative properties of radicals generated by the decomposition of the peroxynitrite-CO_2_ adduct. The results shown in Figure 8 indicate that pheomelanin is equally effective in protecting tyrosine from the nitrative and oxidative action of peroxynitrite also in the presence of 25 mM bicarbonate.

## 3. Discussion

The results of this study show that the photoproperties of pheomelanin can be modulated by various experimental conditions, ranging from the photoprotection to the triggering of potentially damaging photochemical reactions. These properties were studied by analyzing the effect of pheomelanin on UVB radiation-induced oxidation/nitration of tyrosine.

UVB irradiation leads, through tyrosyl radical intermediate, to the dimerization of tyrosine with the formation of 3,3′-dityrosine and in the presence of nitrite the photochemical reaction forms 3-nitrotyrosine as an additional product. The mechanism underlying the formation of 3-nitrotyrosine likely involves the combination of tyrosyl radical with nitrogen dioxide radical (^•^NO_2_), which may be generated by photooxidation of nitrite [53,54]. In the presence of pheomelanin, tyrosine is dose-dependently protected from oxidation to 3,3′-dityrosine both at pH 5.5 and physiological pH (pH 7.4). Furthermore, pheomelanin can perform a protective function on the conversion of tyrosine to 3-nitrotyrosine at pH 7.4. It is known that UVB radiation induces the formation of oxyradicals capable of triggering oxidative reactions [55]. Therefore, the protective action of pheomelanin against the photooxidation of tyrosine could be related to its ability to act as a free radical scavenger.

The experiments conducted on the formation of 3,3′-dityrosine and 3-nitrotyrosine induced by peroxynitrite (ONOO^−^) confirm this hypothesis. Peroxynitrite, which is generated in vivo from the reaction of nitric oxide (^•^NO) with the superoxide anion (O_2_^•−^), is a very reactive species capable of nitrating and oxidizing tyrosine. This reactivity is mediated by the hydroxyl radical (^•^OH) and by the nitrogen dioxide radical (^•^NO_2_) which are generated by the homolytic cleavage of peroxynitrite. In the presence of carbon dioxide (CO_2_), a peroxynitrite-CO_2_ adduct is formed which generates a further radical, the carbonate radical anion (CO_3_^•−)^. Pheomelanin showed protective properties both on the formation of 3,3′-dityrosine and on the conversion of tyrosine to 3-nitrotyrosine induced both by peroxynitrite and peroxynitrite-CO_2_ adduct. These results indicate that pheomelanin can act as free radical scavenger and the observed protective action of the pigment on UVB-induced tyrosine modifications can be attributed to this property.

An interesting result that emerged from our investigations is that pheomelanin can have pro-oxidant properties under some experimental conditions. We observed that the nitration of tyrosine to 3-nitrotyrosine induced by UVB radiation in presence of nitrite at pH 5.5 is increased when carried out in the presence of pheomelanin. These results indicate that the properties of pheomelanin can be significantly influenced by the pH during UVB irradiation, switching from antioxidant (pH 7.4) to pro-oxidant (pH 5.5).

The photochemical experiments conducted with the addition of iron ion are also of particular interest. Pheomelanin has a remarkable ability to bind metals and this property leads often to a modification of the photoprotective capabilities of the pigment. In our experimental conditions by adding Fe(III), we observed a reduced ability to inhibit the oxidative reaction. These results indicate that the antioxidant properties of pheomelanin are sensitive to the effect of metal ions such as iron. It is plausible that the pigment bond with iron induces an increase in the production of highly oxidizing reactive species whose action can only be partially counteracted by the antioxidant activity of the pigment itself.

The production of reactive oxygen species (ROS) resulting from the interaction of oxygen with pheomelanin exposed to UV radiation has often been interpreted as the cause of its pro-oxidant properties. In these hypotheses, pheomelanin (Pheo) would act as a sensitizer and its ability to stimulate the formation of 3-nitrotyrosine (NO_2_Tyr) in the pheomelanin/nitrite/UVB system could be rationalized with the following sequence of reactions:

Pheo → ^1^Pheo*

^1^Pheo* → ^3^Pheo*

^3^Pheo* + O_2_ → Pheo^•+^ + O_2_^•−^

2O_2_^•−^ + 2H_2_O → O_2_ + H_2_O_2_ + 2OH^−^

Fe^3+^ + O_2_^•−^ → Fe^2+^ + O_2_

Fe^2+^ + H_2_O_2_ → Fe^3+^ + ^•^OH + OH^−^

Tyr + ^•^OH → Tyr^•^ + OH^−^

NO_2_^−^ + ^•^OH → ^•^NO_2_ + OH^−^

Tyr^•^ + ^•^NO_2_ → NO_2_Tyr

In competition with the nitrogen dioxide radical (^•^NO_2_), the tyrosyl radical can dimerize with the formation of 3,3′-dityrosine (Dityr):

Tyr^•^ + Tyr^•^ → Dityr

Another possible pathway of formation of the tyrosyl radical and of the nitrogen dioxide radical, responsible for the production of 3-nitrotyrosine, could occur through the direct interaction of the photoexcited pheomelanin in the excited triplet state (^3^Pheo*) with tyrosine and with nitrite (type I mechanism):

^3^Pheo* + Tyr → Pheo^•−^ + Tyr^•^

^3^Pheo* + NO_2_^−^ → Pheo^•−^ + ^•^NO_2_

Photooxidation of pheomelanin-dependent tyrosine can also be mediated by singlet oxygen that is generated by energy transfer from photoexcited pheomelanin in the triplet state to ground state oxygen according to the following scheme (type II mechanism):

^3^Pheo* + O_2_ → ^1^O_2_ + Pheo

^1^O_2_ + Tyr → Tyr^•^ + O_2_^•−^

Tyrosyl radicals can either dimerize or react with a nitrite-derived species to form 3-nitrotyrosine. To our knowledge, the production of nitrating species by direct interaction of nitrite with singlet oxygen has not been reported. It is possible that indirect oxidation of nitrite by the radicals produced by type II mechanism can give rise to further oxidizing species which, as shown above, can contribute to the formation of 3-nitrotyrosine.

The investigations carried out to obtain information on the mechanism through which the nitrite/pheomelanin/UVB system induces the nitration of tyrosine at pH 5.5 indicate that, in our experimental conditions, the process can involve singlet oxygen, indeed, in the presence of D_2_O, the production of 3-nitrotyrosine is considerably higher than that formed in H_2_O. Accordingly, the inhibition exerted by sodium azide on the generation of 3-nitrotyrosine possibly obeys to the known quenching effect on singlet oxygen [21]. These investigations are in agreement with previous studies on nitrite-induced nitration of tyrosine in the presence of methylene blue as a photosensitizer [37]. On the other hand, previous studies carried out in our laboratory have shown that the nitration of tyrosine in presence of riboflavin as a photosensitive agent is mainly of type I [38]. It cannot be excluded that in our case, which uses pheomelanin as photosensitizer, the type I mechanism may participate in the photonitration reaction of tyrosine at slightly acid pH. UVB radiation exposure experiments under anaerobic conditions are currently underway to verify the role of the type I photochemical reaction.

Noteworthy, the photosensitizing effect exerted by pheomelanin is particularly efficient in a slightly acid environment and in the presence of metal ions, such as iron. These conditions, although not physiological, could acquire significance in some pathological situations such as during inflammatory processes or in the case of tissue ischemia. In both these states, pH values close to those used in our experiments are found in vivo (pH 5.8–6.1). Moreover, the forearm of a healthy man has an average surface pH around 5.4–5.9 [56]. Interestingly, pheomelanin synthesis is chemically promoted by weakly acid pH [24,57]. It has been reported that melanosomal pH regulates eumelanin/pheomelanin ratio in melanocytes with a shift towards a pheomelanic phenotype by lowering pH [58,59]. In our experimental conditions, supraphysiological concentrations of nitrite (10 mM) were used to highlight the nitration reaction of tyrosine. Nitrite is the main product of nitric oxide (^•^NO) catabolism, the production of which increases in inflammation [60]. Nitrite is also a constituent of sweat, where it can reach concentrations in the μM range following its formation on the surface of the skin by commensal bacteria. Furthermore, in normal conditions of exposure to the sun and heat, the surface layer of sweat undergoes a rapid increase in concentration caused by evaporation, so that the local concentration of nitrite can increase several times [61].

The result presented herein indicates that photoproperties of pheomelanin can be modulated by various experimental conditions. It is well-known that pheomelanin undergoes structural modifications by UV rays. In the course of the biosynthetic pathways, modification involves benzothiazine units which are gradually converted to benzothiazole [4]. The relative ratio of these two types of pheomelanin moieties appears important in determining whether pheomelanin acts as a pro-oxidant [5,62]. Recently, exploring the photoreactivity of pheomelanin by UVA radiation, the benzothiazole moiety has been shown to be more reactive than benzothiazine moiety [63]. Under our experimental conditions, UVB radiation and reactive nitrogen species could similarly influence pigment photoreactivity and induce structural modifications of pheomelanin worth to be further explored.

## 4. Materials and Methods

### 4.1. Chemicals

l-Cysteine, l-Dopa, l-Tyrosine, diethylenetriaminopentacetic acid (DTPA), sodium azide (NaN_3_), mushroom tyrosinase, horseradish peroxidase was provided by Sigma Aldrich (St. Louis, MO, USA). Deuterium oxide (D_2_O) was obtained from Aldrich (Milwaukee, WI, USA). The 3-nitrotyrosine was from Fluka (Buchs, Switzerland). All other reagents were used with the highest level of purity commercially available. The synthesis of 3,3’-dityrosine was enzymatically carried out from l-Tyrosine and hydrogen peroxide by horseradish peroxidase [64]. Peroxynitrite was prepared from K-nitrite and hydrogen peroxide under acid conditions as previously reported [65].

### 4.2. Synthesis of Pheomelanin

Pheomelanin was synthesized from l-cysteine and l-Dopa [66]. l-Dopa (25 µmoles) was dissolved in 20 mL of K-phosphate buffer (0.05 M pH 6.8) and incubated with mushroom tyrosinase (1.2 mg). After 30 s, l-cysteine (53 μmol) was added, and the reaction mixture was left overnight at 37 °C in agitation. To ensure total conversion to cysteinyldopa isomers, pheomelanin was prepared with cysteine/Dopa molar ratio of 2:1 [67]. The reaction was stopped by reducing pH to 2.2 with 6 N HCl, and the reaction mixture was subsequently centrifuged at 5000 rpm for 25 min. The residue is suspended in H_2_O and subsequently lyophilized, thus obtaining approximately 3.4 mg of dark brown pheomelanin. The identification of pheomelanin as a synthetic product was performed by analyzing the absorption spectrum (λ = 800–200 nm) of a solution containing 2.5 μg/mL pheomelanin in 1 M K-phosphate buffer, pH 8.0. The absorbance of a 4 μg/mL pheomelanin solution was on average 0.092 at 400 nm [68]. Stock solutions of synthetic pheomelanin were in 1 M K-phosphate buffer, pH 8.0. The spectrum analysis was carried out using a UV-vis Cary 50 Scan spectrophotometer.

### 4.3. Nitration and Oxidation of l-Tyrosine Induced by UVB Radiation

The reaction mix containing 1 mM l-Tyrosine, 0.05 M K-phosphate buffer, pH 7.4, or 0.2 M K-phosphate, pH 5.5 and 0.1 mM DTPA, was incubated in the absence or by adding 10 mM K-nitrite to a Petri dish. To study the effect of pheomelanin on the oxidative/nitrative modifications of tyrosine induced by UVB rays, the final concentration of the pigment in the reaction mixture was 4.2 μg/mL. The photooxidation of tyrosine was initiated by exposing the reaction mixture to UVB radiation produced by two fluorescent lamps at room temperature. The irradiation was interrupted for 1 min every 5 min to mix the suspension and prevent overheating of the reaction mixture. After 30 min of irradiation, the samples were centrifuged for 5 min at 12,000 rpm, and the supernatant analyzed by HPLC to verify the formation of 3,3’-dityrosine and 3-nitrotyrosine.

### 4.4. Exposure to UVB Radiation

The exposure was carried out in an irradiation cabin built by the Bioltecnical Service, Nettuno, RM (Italy). Two Sankyo Denki G15T8E UVB fluorescent lamps (λ = 270–320 nm with a maximum peak at 313 nm) were mounted on the ceiling of a closed aluminum cabin equipped with a front door for loading the Petri dishes. The lamps emit a luminous flux, with energy administered in the unit of time equal to 2.5 J m^−2^s^−1^, perpendicular to the radiation plane placed at 23 cm distance. The total energy administered was 4500 J m^−2^. The lamps have an efficiency equal to 1, i.e., all the absorbed power is transformed into UV radiation; furthermore the radiation emitted, given the geometry of the cabin and the reflectivity of the walls, ends entirely on the irradiation plane.

### 4.5. Nitration and Oxidation of l-Tyrosine Induced by Peroxynitrite

The experiments with peroxynitrite were performed as described in [69]. The reaction mixture containing 4.2 μg/mL pheomelanin, 100 μM l-Tyrosine, 0.2 M K-phosphate buffer, pH 7.4 and 0.1 mM DTPA, was incubated in the absence or presence of 25 mM Na-bicarbonate. The reaction was started by the addition of peroxynitrite (final concentration of 100 μM). After 5 min at room temperature, the solution was centrifuged for 5 min at 12,000rpm, and the supernatant analyzed by HPLC to verify the formation of 3-nitrotyrosine and 3,3’-dityrosine.

### 4.6. HPLC Analysis

3-nitrotyrosine and 3,3’-dithyrosine were analyzed by HPLC using a Waters chromatograph equipped with a model 600 pump and a model 600 gradient control module as reported [65,70]. Chromatographic separation was performed using a Nova-pak column. C18 (3.9 mm × 150 mm), 4 μm (Waters) and as mobile phase: (A) K-phosphate/H_3_PO_4_ buffer, 50 mM, pH 3.0; (B) acetonitrile-water (50:50, *v*/*v*) with a flow rate of 1 mL/min at room temperature and a linear gradient from A to 33% of B in 10 min. 3-nitrotyrosine was analyzed at 360 nm, using a Waters 996 photodiode spectrophotometric detector. 3,3’-dithyrosine was analyzed using a Waters 474 fluorescence detector, setting the wavelength at 260 nm for the excitation and at 410 nm for emission. Peaks were identified using external standards and sample concentrations were calculated using standard curves. The elution times of 3-nitrotyrosine and 3,3’-dithyrosine are 8.9 and 7.5 min, respectively. The limit of determination of 3-nitrotyrosine and 3,3’-dithyrosine is 20 pmoles and 1 pmol, respectively.

### 4.7. Data Analysis

The results are expressed as mean values ± SEM of at least three separate experiments performed in duplicate. The statistical analyses were performed using Student’s *t*-test; *p* < 0.05 was deemed significant. The graphs and data analysis were performed using the GraphPad Prism 4 program.

## 5. Conclusions

UVB radiation induces the photooxidation/photonitration of tyrosine. Pheomelanin is able to perform a protective function both on the tyrosine oxidation to 3,3′-dityrosine and on the conversion of tyrosine to 3-nitrotyrosine when the exposure is conducted at physiological pH; conversely at pH 5.5, the presence of pheomelanin induces a 60% increase in the formation of 3-nitrotyrosine. The photosensitizing action of pheomelanin in the nitration reaction of tyrosine to 3-nitrotyrosine at pH 5.5, is further increased by about 8 times in D_2_O, suggesting a role of ^1^O_2_ in the reaction mechanism. The addition of Fe(III) during the irradiation of tyrosine in presence of nitrite provokes a decrease of the antioxidant activity of pheomelanin also against the formation of 3,3’-dityrosine, indicating that the photoproperties of pheomelanin may be affected by the presence of metal ions. Finally, pheomelanin showed protective properties on oxidation/nitration of tyrosine induced by peroxynitrite and by the decomposition of the peroxynitrite-CO_2_ adduct. An important implication of the results obtained is that the pheomelanin-dependent photonitration of tyrosine in presence of nitrite could exert toxic effects by inducing the nitration of tyrosine protein residues present in the skin with consequent functional alteration of proteins themselves.

## Data Availability

Not applicable.

## Supplementary Materials

The following are available online at https://www.mdpi.com/article/10.3390/ijms23010267/s1.
