# Supplementary material for: Pheomelanin Effect on UVB Radiation-Induced Oxidation/Nitration of l-Tyrosine
Source: Int J Mol Sci. 2021 Dec 27;23(1):267. doi: 10.3390/ijms23010267 (PMC8745751; doi:10.3390/ijms23010267)

Figure S1. Synthetic pheomelanin (2.5 μg/mL) has been dissolved in 1 M K-phpsphate buffer, pH 8.0

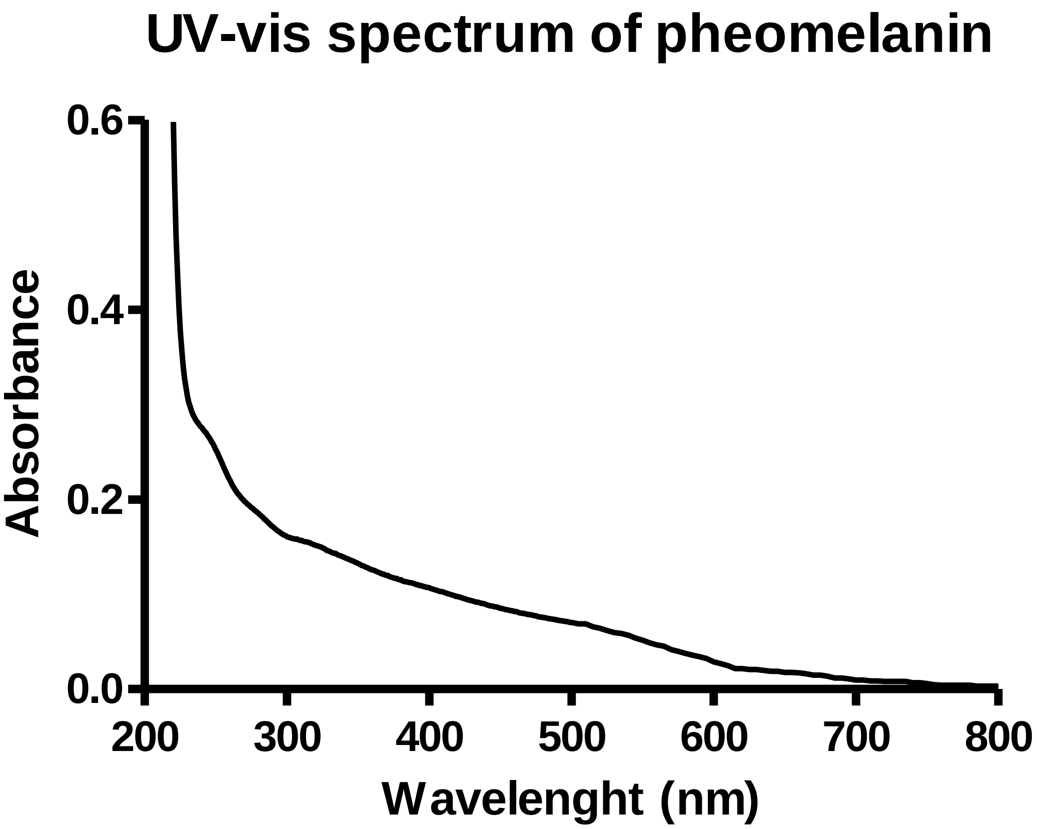

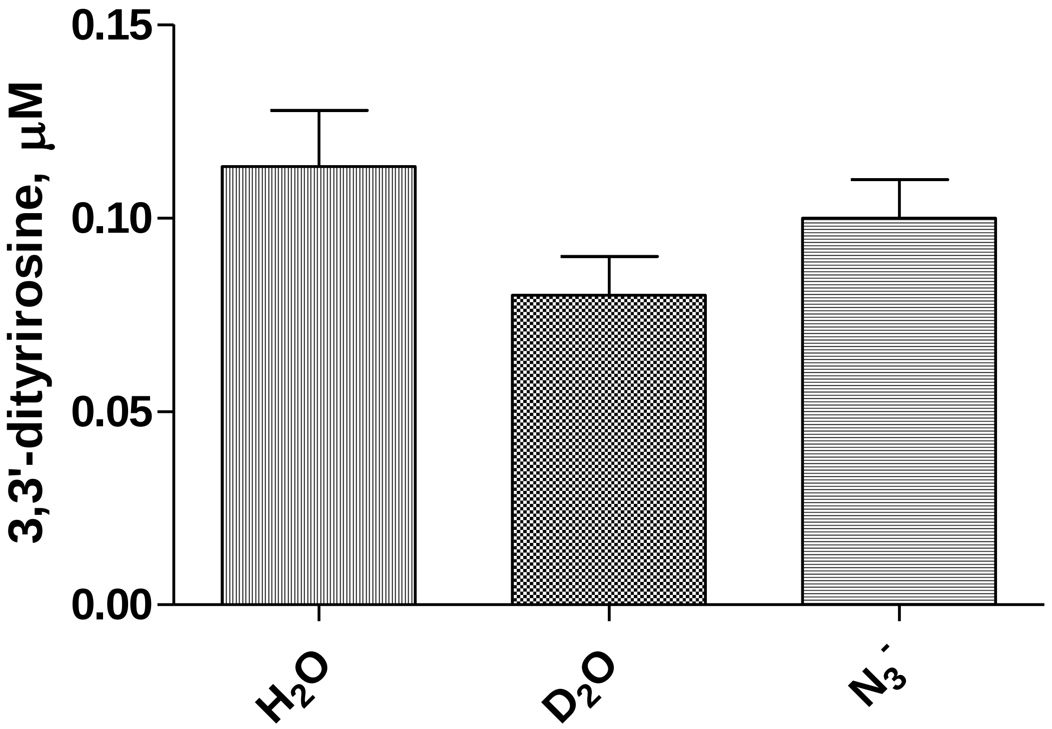

Figure S2. Photooxidation of tyrosine by the nitrite/pheomelanin/UVB system: effect of D_2O and NaN_3.
Pheomelanin 4.2 μg/mL is added to the solution, containing 1 mM tyrosine, 10 mM K-nitrite in 0.2 M K-phosphate buffer at pH 5.5 and 0.1 mM DTPA. The solution is exposed to UVB rays for 30 minutes. The reaction is stopped by placing the mixture in the dark and the supernatant, obtained after centrifugation, is analyzed by HPLC to determine the formation of 3,3'-dityrosine, as reported in Materials and Methods. In D_2O, the pD (5.5) was taken as the measured pH + 0.4. NaN_3 is added to a final concentration of 1 mM.

Figure S3. Eumelanin effect on peroxynitrite-induced dityrosine and nitrotyrosine formation
To the reaction mix containing 100 µM tyrosine, 4.0 µg/mLeumelanin, 0.1 mM DTPA in 0.2 M K-phosphate buffer, 100 µM peroxynitrite is added, Na-bicarbonate when present is at a concentration of 25 mM. After 5 minutes at room temperature, the reaction mixture is analyzed by HPLC to measure 3,3'-dityrosine and 3-nitrotyrosine, as reported in Materials and Methods. *$p < 0.05$

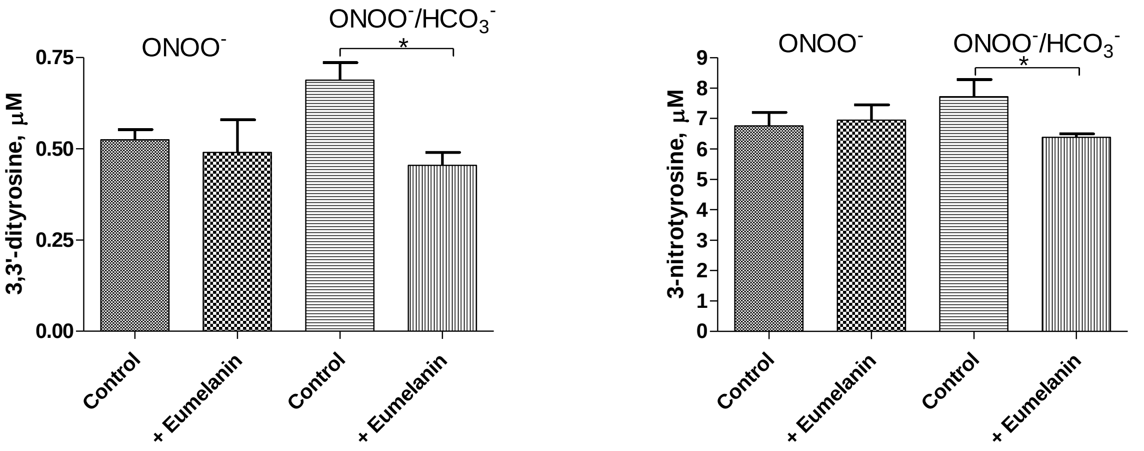

Supplement: Supplementary file 1 [file ijms-23-00267-s001.zip › ijms-1509342-supplementary.pdf]
